# Associations between single and combined occupational mechanical exposures and surgery for subacromial impingement syndrome: a nationwide Danish cohort study

**DOI:** 10.5271/sjweh.4032

**Published:** 2022-08-31

**Authors:** Annett Dalbøge, Poul Frost, Johan Hviid Andersen, Susanne Wulff Svendsen

**Affiliations:** 1 Danish Ramazzini Centre, Department of Occupational Medicine, Aarhus University Hospital, Aarhus, Denmark; 2 Danish Ramazzini Centre, Department of Occupational Medicine - University Research Clinic, Gødstrup Hospital, Denmark

**Keywords:** acromioplasty, duration, intensity, JEM, job exposure matrix, shoulder disorder, work

## Abstract

**Objective:**

This study aimed to evaluate whether the risk of surgery for subacromial impingement syndrome (SIS) increases with the number of combined occupational mechanical exposures compared with single exposure.

**Methods:**

We reanalyzed data from a register-based cohort study of the entire Danish working population (N=2 374 403) with 14 118 events of surgery for SIS (2003–2008). Exposure information in 10-year windows was obtained by combining occupational codes with a job exposure matrix. For single and combined mechanical exposures, we created three exposure variables of the number of years with specific exposure intensities with or without co-existing mechanical exposures. We used logistic regression as survival analysis.

**Results:**

We found exposure–response relations for duration and intensity of each single mechanical exposure except for repetition. The single effect of arm elevation >90º reached a maximum adjusted odds ratio (OR_adj_) of 1.7 [95% confidence interval (CI) 1.5–2.0], which increased to 1.8 (95% CI 1.5–2.0), 2.0 (95% CI 1.9–2.2), and 2.2 (95% CI 2.0–2.5) when combined with repetition, force, and both. When combining repetition with arm elevation >90º, force, and both, OR_adj_ increased from 1.5 (95% CI 1.3–1.8) to 2.1 (95% CI 1.8–2.4), 2.5 (95% CI 2.4–2.9), and 2.7 (95% CI 2.4–3.0). For force, OR_adj_ increased from 2.5 (95% CI 2.1–2.9) to 2.6 (95% CI 2.3–2.8), 2.8 (95% CI 2.4–3.2), and 3.0 (95% CI 2.6–3.4).

**Conclusion:**

We found an increased risk of surgery for SIS with the number of combined exposures; the risk was especially pronounced when the combined exposures included force.

Occupational mechanical exposures such as working with upper arm elevation, repetitive shoulder movements, and forceful shoulder exertions (eg, lifting, carrying, pushing, and pulling loads) are considered risk factors for clinically diagnosed subacromial impingement (SIS) (1–5) and surgery for SIS (6–13). Exposure to hand-arm vibrations (HAV) is less well studied (7–9, 14–20). To examine associations between occupational mechanical exposures and SIS, we have conducted several epidemiological studies using a nationwide cohort of almost 2.5 million people (7–10, 13) and a job exposure matrix (’The Shoulder JEM’) (6, 7, 21). In three of these studies, we showed that the risk of surgery for SIS is related to cumulative exposures accrued over a 10-year exposure time window (7, 8, 10). However, we did not control for co-existing occupational mechanical exposures due to the high correlations between the exposures. A new analytical approach enabled us to evaluate safe exposure intensities that do not entail an increased risk even after prolonged exposure duration (9, 13). We found indications of safe exposure intensities for repetition (median angular velocity <45 ˚/s), while arm elevation >90º (>2.25 minutes/day), forceful shoulder exertion (≥10% of maximal voluntary electrical activity), lifting/carrying loads ≥10 kg (>0.0 times/day), and pushing/pulling loads ≥50 kg (>0.0 times/day) implied an increased risk even with minimal exposure when assessed across 10-year exposure time windows (9, 13). No exposure-response relation was found for HAV (9). In these analyses, we were able to adjust for the cumulative effect of other occupational mechanical exposures, indicating that mechanical exposures comprising arm elevation >90º, repetitive shoulder movements, forceful shoulder exertions (eg, lifting/carrying and pushing/pulling loads) can be considered independent risk factors for surgery for SIS.

In systematic reviews, strong evidence of an association between combined mechanical exposures and SIS has been found (1–3). However, it is difficult to compare the effect of the different combinations of mechanical exposures and to differentiate between the contribution of each exposure due to different study populations (eg, reference groups) and different outcome definitions and because mechanical exposures have been defined and assessed heterogeneously across studies. Our national cohort and The Shoulder JEM enabled us to overcome these challenges. This study addressed the hypothesis that the risk of surgery for SIS increases with the number of combined exposures compared with single exposure.

## Methods

### Design and study population

We used data from a previous cohort study of the entire Danish working population (7). The cohort has previously been described (7). In brief, the cohort included 2 374 403 persons born in Denmark between 1933 and 1977, living in Denmark in 2003, with ≥5 years of full-time employment between 1993 and 2007, and no previous shoulder surgery between 1996 and 2002 (7). The cohort was followed from 2003 to 2008. The Danish Data Protection Agency approved the study (j. no.: 2012-41-1187). In Denmark, register studies do not need approval from the Committee System on Biomedical Research Ethics (request no. 130/2009). The reporting of the study follows the STROBE guideline on observational studies (22).

### Outcome

The outcome was first-time surgery for SIS defined as a SIS-related principal diagnosis (International Classification of Diseases 10^th^ revision; M75.1-M75.9 or M19, without a secondary diagnosis of M75.0) and a SIS-related surgery code (Nordic Medico Statistical Committee´s classification of surgical procedures; KNBA, KNBE-H, and KNBK-M) (7). Information on the outcome was obtained from The Danish National Patient Register (23, 24).

### Exposures

Occupational mechanical exposures included working with arm elevation >90°, repetitive shoulder movements, and forceful shoulder exertions. Exposure estimates for 10-year exposure time windows were obtained for each cohort member by combining individual information on work history with The Shoulder JEM (7, 9). Individual year-by-year information on work history between 1993 and 2008 was obtained using the Danish version of the International Classification of Occupations from 1988 (D-ISCO 88) from the Employment Classification Module (ECM) (25). Each cohort member’s D-ISCO 88 codes were combined with The Shoulder JEM (6, 7, 21). The Shoulder JEM comprises all D-ISCO 88 codes divided into 172 expected homogenous job groups cross-tabulated with the intensity of arm elevation >90°, repetitive shoulder movements, forceful shoulder exertions, and HAV. Originally, the exposure intensity in The Shoulder JEM was based on expert ratings. Five occupational health physicians rated each occupational mechanical exposure for each job group based on what they would expect to reach from a critical interview with a typical employee (7, 26). For arm elevation >90°, the experts were asked “For the given job group, how many hours does a typical employee work with one or both elbows above shoulder height?” The mean of the five experts’ ratings were included in The Shoulder JEM. For arm elevation >90° and repetitive shoulder movements, the expert rated exposure estimates were calibrated into predicted measured job exposures using approximately 500 whole day technical measurements (inclinometry) (21). In this study, we used the predicted measured job exposures for time spent with arm elevation >90° (min/day) and repetitive shoulder movements in terms of angular velocity (°/s), and the expert ratings of forceful shoulder exertions [a five-point rating of intensity of exertion (0-4)] (6, 7). No job groups in The Shoulder JEM were rated with an intensity of 4 (“near maximal”).

We adjusted the exposure estimates according to the weekly working hours using the following factors: 1 (≥37 hours/week), 0.75 (≥28 to <37 hours/week), 0.5 (≥18.5 to <28 hours/week), 0.25 (≥9 to <18.5 hours/week), and 0.0 (<9 hours/week) (7).

For each single mechanical exposure, we created three intensity-specific exposure duration variables. The three variables were created based on the distribution of the exposure intensity to ensure large exposure groups with exposure contrast (9). For arm elevation, the categories were >2.25–5.00, ≥5.00–10.00, and ≥10.00–30.00 min/day, for repetition >27–35, ≥35–45, and ≥45–70 º/s, and for force >0.0–0.5, ≥0.5–1.5, and ≥1.5–3.0 (9). To study the effect of ≥2 combined mechanical exposures, we created intensity-specific exposure duration variables as described above but in combination with one or two co-existing mechanical exposures above minimal intensity. For example, each of the three categories of arm elevation were combined with repetition >27 º/s, force >0, and both of these (repetition >27 º/s and force >0). Likewise, each of the three categories of repetition were combined with arm elevation >2.25 min/day, force >0, and both of these. Force was combined with arm elevation >2.25, repetition >27 º/s, and both. This means that eg, the analyses of arm elevation (>2.25–5.00, ≥5.00–10.00, and ≥10.00–30.00 min/day) combined with repetition (>27 º/s) differ from the analyses of repetition (>27–35, ≥35–45, and ≥45–70 º/s) combined with arm elevation (>2.25 min/day). Exposure duration was calculated by summing up the number of exposure years with each single or combined exposure intensity in each 10-year exposure time window (the number of years could range from 0–10). An example of estimating the exposure duration for arm elevation single and arm elevation combined with force >0 is shown in the supplementary material, www.sjweh.fi/article/4032, table S1.

### Covariates

A priori, we decided to include register-based information on age, sex, region of residence, calendar year at start of follow up, and number of the specific follow-up year as covariates (7, 9, 10, 13). To evaluate the effect of a single mechanical exposure, we calculated 10-year cumulative exposure estimates using the pack-year concept of smoking (ie, arm elevation-years, repetition-years, force-years, and HAV-years) (7, 9). Information on expert-based HAV (min/day) was obtained from The Shoulder JEM. To evaluate the effect of two combined exposures (eg, arm elevation combined with force), we also constructed two exposure variables, which counted number of years with either exposure (eg, arm elevation >2.25 min/day without force (force=0) and force >0 without arm elevation (arm elevation=2.25 min/day) (disregarding repetition). Similarly, when evaluating the effect of three combined exposures, we additionally constructed an exposure variable, which counted the number of years with two exposures without the third.

### Statistical analyses

For single and combined exposures, we performed pairwise correlation analyses between the three intensity-specific exposure duration variables. We used a logistic regression technique equivalent to discrete survival analysis (27) with time-varying exposures and a one-year lag (7, 9, 10, 13). The statistical unit was person-years (27). To evaluate the effect of a single exposure, we repeated the analysis performed in a previous study including the three intensity-specific exposure duration variables (11 categories, 0–10 years), age (five categories), sex, region of residence (five categories), calendar year at start of follow up (continuous), number of the specific follow-up year, and the cumulative exposures to the other mechanical exposures (eg, arm-years, repetition-years, force-years, and HAV-years) (9). When estimating the effect of two combined exposures, we additionally adjusted for the two covariates that counted the number of years with single exposures to the types of exposure in focus (eg, for the combination of arm elevation and force, the two relevant single exposures were arm elevation without force and force without arm elevation, disregarding repetition). When estimating the effect of three combined exposures, we further adjusted for the covariate that counted the number of years with single exposure and the covariate that counted the number of years with two exposures. All analyses included all person-years. Tests for trend were performed with the intensity-specific exposure duration variables in continuous versions. To test the robustness of our results, we changed the cut-off values for each of the three intensity-specific exposure duration variables (eg, for arm elevation combined with force, the lowest exposure group was changed from >2.25–5.00 to >2.25–4.50 and >2.25–5.50 min/day. The analyses were performed on Statistics Denmark’s research platform using STATA 16 (Stata Corp, College Station, TX, USA).

## Results

Flow chart and descriptive characteristic of the study population have been presented previously (7, 9). During follow-up, 14 118 first-time events of surgery for SIS occurred. For each single mechanical exposure, low correlation coefficients were found for the three intensity-specific exposure duration variables with correlation coefficients -0.26–0.06 (9). The correlation coefficients for the three durations of arm elevation with specific exposure intensities combined with repetition, force, and both were -0.14– -0.07, -0.09– -0.07, and -0.12– -0.07, respectively (supplementary table S2. For repetition combined with arm elevation, force, and both, the correlation coefficients were -0.14– -0.04, -0.13– -0.04, and -0.12– -0.04, respectively. For force combined with arm elevation, repetition, and both, the correlation coefficients were -0.24– -0.05, -0.25– -0.10, and -0.12– -0.05, respectively.

**Figure 1:**
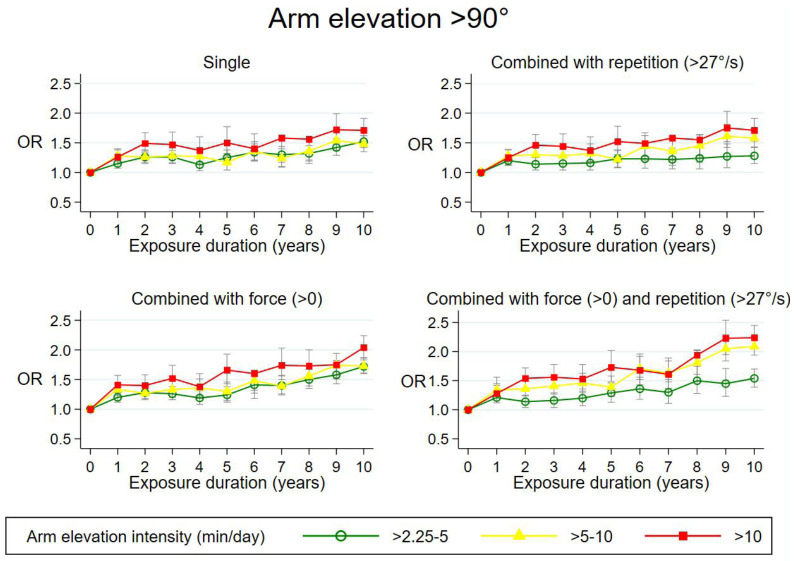
Adjusted odds ratios (OR)* with 95% confidence intervals of surgery for subacromial impingement syndrome in relation to duration of exposure (years) at different arm elevation intensities; single or combined with other mechanical exposures across 10-year exposure time windows. *Single*: Adjusted for the other two arm elevation duration variables, age, sex, region of residence, calendar year at start of follow up, number of follow-up years, and cumulative effects of three other mechanical exposures including hand-arm vibrations. *Combined*: Adjusted for the other two arm elevation duration variables, age, sex, region of residence, calendar year at start of follow up, number of follow-up years, cumulative exposures of one or two other mechanical exposures including hand-arm vibrations and the 2 variables, which counted years of exposure with one specific mechanical exposure occurring without the other.

**Figure 2:**
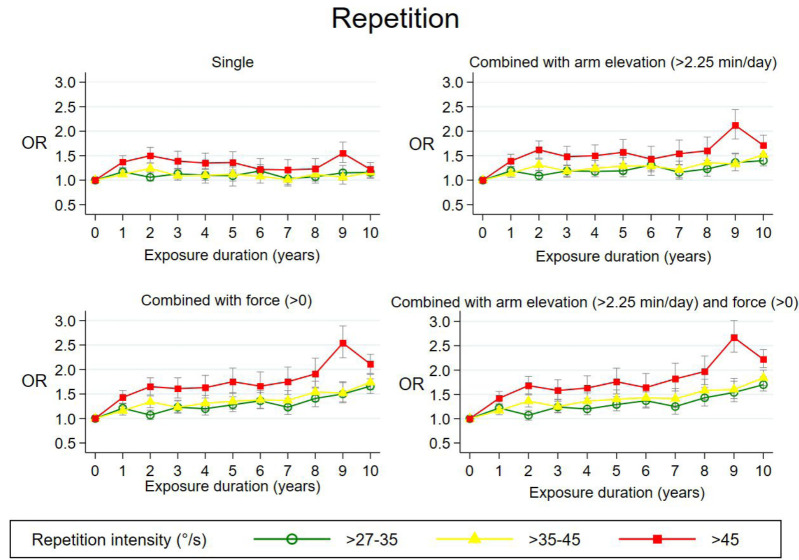
Adjusted odds ratios (OR)* with 95% confidence intervals of surgery for subacromial impingement syndrome in relation to duration of exposure (years) at different repetition intensities; single or combined with other mechanical exposures across 10-year exposure time windows. *Single:* Adjusted for the other two repetition duration variables, age, sex, region of residence, calendar year at start of follow up, number of follow-up years, and cumulative effects of three other mechanical exposures including hand-arm vibrations. *Combined*: Adjusted for the other two repetition duration variables, age, sex, region of residence, calendar year at start of follow up, number of follow-up years, cumulative exposures of one or two other mechanical exposures including hand-arm vibrations and the two variables, which counted years of exposure with one specific mechanical exposure occurring without the other.

**Figure 3:**
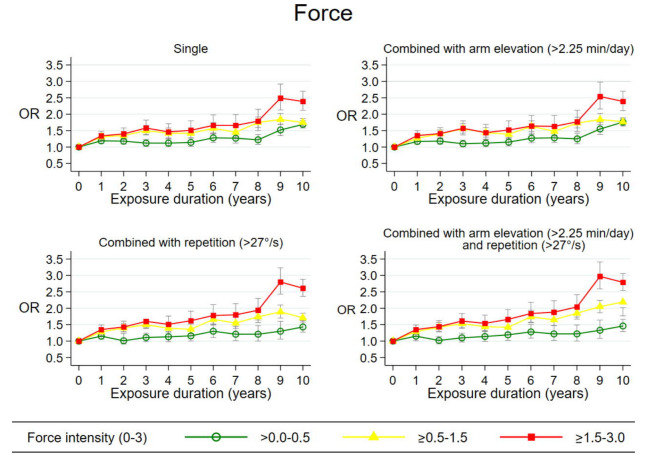
Adjusted odds ratios (OR)* with 95% confidence intervals of surgery for subacromial impingement syndrome in relation to duration of exposure (years) at different force intensities; single or combined with other mechanical exposures across 10-year exposure time windows. *Single*: Adjusted for the other two force duration variables, age, sex, region of residence, calendar year at start of follow up, number of follow-up years, and cumulative effects of three other mechanical exposures including hand-arm vibrations. *Combined*: Adjusted for the other two force duration variables, age, sex, region of residence, calendar year at start of follow up, number of follow-up years, cumulative exposures to one or two other mechanical exposures including hand-arm vibrations, and the two variables, which counted years of exposure with one single mechanical exposure occurring without the other.

Figures 1–3 show exposure–response relations for all three single exposures and in combination with other mechanical exposures across the 10-year time-window. The three intensities are represented by green (low intensity), yellow (medium intensity), and red curves (high intensity).

*Arm elevation*. Based on figure 1, we found exposure–response relations for durations of arm elevation at all three exposure intensities when controlling for the cumulative effect of repetition, force, and HAV, reaching a maximum adjusted odds ratio (OR_adj_) of 1.7 [95% confidence interval (CI) 1.5–2.0] (9). When combining arm elevation with repetition >27 °/s, arm elevation with force >0, and arm elevation with both repetition and force, maximum OR_adj_ of 1.8 (95% CI 1.5–2.0), 2.0 (95% CI 1.9–2.2), and 2.2 (95% CI 2.0–2.5) were found. All P-values for tests for trend were <0.000. When combining arm elevation with force, the mean force intensities were 0.6, 0.8, and 1.3 for the three intensities of arm elevation combined with force. In the robustness analyses, small changes in exposure cut-off values showed no overall change in OR_adj_. The figure can be used to calculate OR for persons with different 10-year exposure profiles. For example, you can calculate the OR for persons with a 10-year time window, which includes three years as minimally exposed with arm elevation single (OR 1.0), two years with an arm elevation intensity >5.00–10.00 and force >0 (yellow curve; OR=1.4), and five years with an arm elevation intensity ≥10.00, force >0, and repetition >27 °/s (red curve; OR=1.7) as the product of the OR for the exposed years, that is 1.0×1.4×1.7=2.38.

*Repetition*. In figure 2, angular velocities of >27–35 and ≥35–45 º/s showed no increasing OR_adj_, while an increased OR_adj_ was found for angular velocities ≥45 º/s with a maximum OR_adj_ of 1.5 (95% CI 1.3–1.8) (9). When combining repetition with arm elevation >2.25 min/day, force >0, or both, the maximum OR_adj_ increased to 2.1 (95% CI 1.8–2.4), 2.5 (95% CI 2.4–2.9), and 2.7 (95% CI 2.4–3.0). P-values for tests for trend were statistically significant (P<0.000) except for single repetition with low intensity. Force intensities were 0.8, 1.0, and 1.4 for the three intensities of repetition combined with force. In the robustness analyses, small changes in exposure cut-off values showed no overall change in OR_adj_.

*Force*. Based on figure 3, we found exposure–response relations for durations of force at all three exposure intensities reaching a maximum OR_adj_ of 2.5 (95% CI 2.1–2.9) (9). The maximum OR_adj_ were 2.6 (95% CI 2.3–2.8), 2.8 (95% CI 2.4–3.2), and 3.0 (95% CI 2.6–3.4) when combining force with arm elevation >2.25 min/day, repetition >27 °/s, and both. All P-values for trend test were statistically significant (P<0.000). Mean intensities of repetitive shoulder movements were 40 °/s, 38 °/s, and 48 °/s for the three intensities of force combined with repetition. Only small changes in OR_adj_ were found in the robustness analyses.

## Discussion

We found exposure–response relations between surgery for SIS and all single occupational mechanical exposures except for repetition <45 °/s (9). When combining mechanical exposures, the risk of surgery for SIS increased with the number of combined exposures; the highest risks were found when combining all three mechanical exposures. Force seemed to increase the risk the most.

We have previously outlined the methodological strengths and limitations of the study design (7, 9, 10, 13, 21). In brief, strengths included a cohort of the entire Danish working population and objective information on exposure, outcome, and potential confounders. Based on information on year-by-year D-ISCO 88 codes and our validated Shoulder JEM, we were able to obtain information of both exposure intensity and duration without recall bias. Our new analytic approach, further enabled us to adjust for co-existing mechanical exposures (ie, duration of exposures at other exposure intensities and cumulative mechanical exposures). A limitation of the study was the restricted number of lifestyle factors due to the register design. In our previous studies, we have adjusted for social class and repeated the analysis for the largest social group (intermediate class) which did not change the results much (7, 9, 13). In a case–control study with cases and controls randomly selected from the cohort, adjusting for self-reported lifestyle factors (eg, smoking, leisure time physical activity, and diabetes mellitus) did not change the results significantly (8). Socioeconomic differences in access to surgery were minimized through the Danish public healthcare system which is financed through taxes.

For both single and combined exposures (eg, arm elevation >2.25–5.00 min/day combined with force >0), employees who in a 10-year time window were more (eg, arm elevation ≥5.00 min/day and force >0) or less exposed (eg, arm elevation=2.25 min/day and force >0) would attain 0 years in the given exposure interval, and therefore would be grouped together as the “reference group”. The low correlation found for the different single and combined exposure variables, allowed us to mutually adjust for other exposure intensities (eg, arm elevation ≥5.00 min/day and force >0 and arm elevation=2.25 min/day and force >0), providing a reference group which represents employees with minimal exposures. When additionally adjusting for the cumulative effect of other mechanical exposures, the reference group represents workers with minimal exposure to arm elevation, repetition, and force. This minimally exposed reference group allowed us to compare results between graphs and thus compare the effects of different mechanical exposures.

In our analyses of the combination of two mechanical exposures, we evaluated the effect of a mechanical exposure (eg, arm elevation >2.25–5.00, ≥5.00–10.00, and ≥10.00–30.00 min/day) in combination with another exposure above minimal (eg, force >0). We were not able to evaluate the effect of a mechanical exposure (eg, arm elevation) combined with higher levels of another exposure (eg, force ≥2.0) due to few observations; arm elevation ≥10.00–30.00 min/day seldom occurs with force >2.0. As all three mechanical exposures often co-occur in occupational settings, we could not generate exposure variables defined by the co-existing of only two exposures (eg, arm elevation and force) without the third exposure (eg, repetition). The data structure only allowed us to control for the third exposure as a cumulative variable.

In our systematic review of the association between occupational mechanical exposures and SIS, we found strong evidence for combined mechanical exposure with measures of association between 0.6 (95% CI 0.26–1.48) and 7.1 (95% CI 1.94–25.66) (3). The existing literature has often evaluated the effect of two combined exposures without adjustments for co-existing mechanical exposures (6–8, 15, 28–32). The results of our study support the association between combined mechanical exposures and SIS after adjusting for co-existing mechanical exposures, and indicate an additional increase in risk with the number of combined exposures. The small increase in risk when combining arm elevation with repetition >27 °/s might reflect that workers with arm elevation do not have repetition above the exposure threshold of 45 °/s. For both arm elevation and repetition, we found that the risk was especially pronounced when combined with force, but the highest risks were found when combining all three mechanical exposures. We are not aware of studies, which have evaluated the combined effects of all three mechanical exposures (ie, arm elevation, repetition, and force), while adjusting for HAV.

Patients with surgery for SIS can be considered to have relatively severe shoulder symptoms. The clinical decision to offer surgery versus non-surgical treatment for SIS might be influenced by the patient’s occupational mechanical exposure, and therefore potentially lead to an overestimation of the association between mechanical exposures and surgery for SIS. However, we found similar results as studies for clinically assessed SIS with OR of 0.6–7.1. This indicates that our results for single and combined mechanical exposures can be generalized to clinically diagnosed SIS and perhaps shoulder pain. The results from this study could probably be extended to other countries similar to Denmark.

### Concluding remarks

In conclusion, we found an additional increase in risk of surgery for SIS with the number of combined mechanical exposures; the risk was especially pronounced when the combined exposures included forceful shoulder exertions. Based on our results, preventive actions should focus on reducing combined exposures with a special focus on forceful shoulder exertions.

## Supplementary material

Supplementary material
