# Supplementary material for: Associations between single and combined occupational mechanical exposures and surgery for subacromial impingement syndrome: a nationwide Danish cohort study
Source: Scand J Work Environ Health. 2022 Aug 31;48(6):490–7. doi: 10.5271/sjweh.4032 (PMC9888441; doi:10.5271/sjweh.4032)
Supplement: Supplementary material [file SJWEH-48-490-S001.pdf]

Associations between single and combined occupational mechanical exposures and surgery for subacromial impingement syndrome: a nationwide Danish cohort study[1]

by Annett Dalbøge, PhD,[2] Poul Frost, PhD, Johan Hviid Andersen, PhD, Susanne Wulff Svendsen, PhD

1. Supplementary material
2. Correspondence to: Annett Dalbøge, Danish Ramazzini Centre, Department of Occupational Medicine, Aarhus University Hospital, Palle Juul-Jensens Boulevard 99, 8200 Aarhus N, Denmark. [E-mail: anetaner@rm.dk].

Table S1. Estimation of exposure duration for arm elevation single and arm elevation combined with force >0 for a 10-year time window with a 1-year lag.

Year	Arm elevation (min/day)	Force (score)	Arm elevation single			Arm elevation combined with force >0		
			>2.25–5.00 min/day	≥5.00–10.00 min/day	≥10.00 min/day	>2.25–5.00 min/day	≥5.00–10.00 min/day	≥10.00 min/day
1993	12.0	2	0	0	1	0	0	1
1994	12.0	2	0	0	1	0	0	1
1995	7.5	1	0	1	0	0	1	0
1996	7.5	1	0	1	0	0	1	0
1997	7.5	1	0	1	0	0	1	0
1998	7.5	1	0	1	0	0	1	0
1999	7.5	1	0	1	0	0	1	0
2000	7.5	1	0	1	0	0	1	0
2001	7.5	0	0	1	0	0	0	0
2002	7.5	0	0	1	0	0	0	0
**-**	**-**	**-**	**0**	**8**	**2**	**0**	**6**	**2**

Table S2. Correlation matrix between the intensity-specific exposure duration variables for the different exposure combinations.

	Low	Medium	High
**Arm elevation combined with repetition**			
Low	1.00		
Medium	-0.10	1.00	
High	-0.14	-0.07	1.00
**Arm elevation combined with force**			
Low	1.00		
Medium	-0.07	1.00	
High	-0.09	-0.07	1.00
**Arm elevation combined with repetition and force**			
Low	1.00		
Medium	-0.10	1.00	
High	-0.12	-0.07	1.00

	Low	Medium	High
**Repetition combined with arm elevation**			
Low	1.00		
Medium	-0.09	1.00	
High	-0.14	-0.04	1.00
**Repetition combined with force**			
Low	1.00		
Medium	-0.09	1.00	
High	-0.13	-0.04	1.00
**Repetition combined with arm elevation and force**			
Low	1.00		
Medium	-0.08	1.00	
High	-0.12	-0.04	1.00

	Low	Medium	High
**Force combined with arm elevation**			
Low	1.00		
Medium	-0.24	1.00	
High	-0.15	-0.05	1.00
**Force combined with repetition**			
Low	1.00		
Medium	-0.12	1.00	
High	-0.10	-0.25	1.00
**Force combined with arm elevation and repetition**			
Low	1.00		
Medium	-0.12	1.00	
High	-0.10	-0.05	1.00
